# Supplementary figures and images for: DuoHexaBody-CD37 induces direct cytotoxic signaling in diffuse large B-cell lymphoma
Source: eLife. 2026 Jul 20;14:RP106425. doi: 10.7554/eLife.106425 (PMC13384490; doi:10.7554/eLife.106425)

Figure 5 – figure supplement 1A

SHP1 Knock out in Oci-Ly7 and HBL1 cell lines

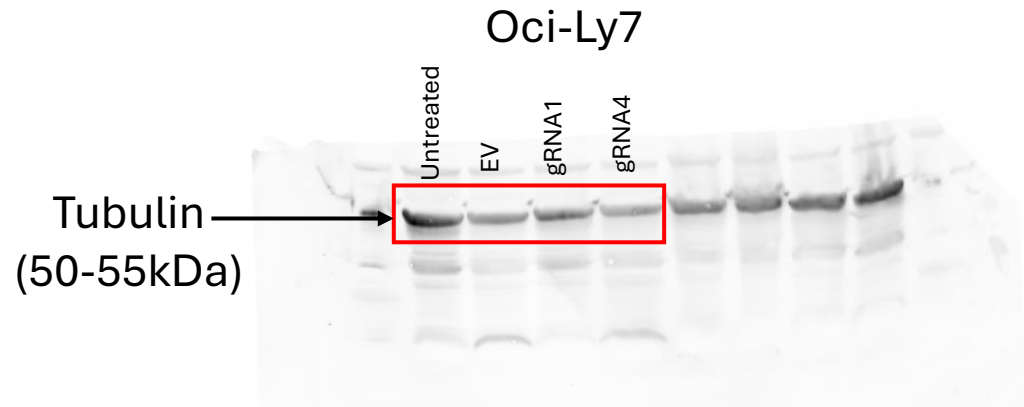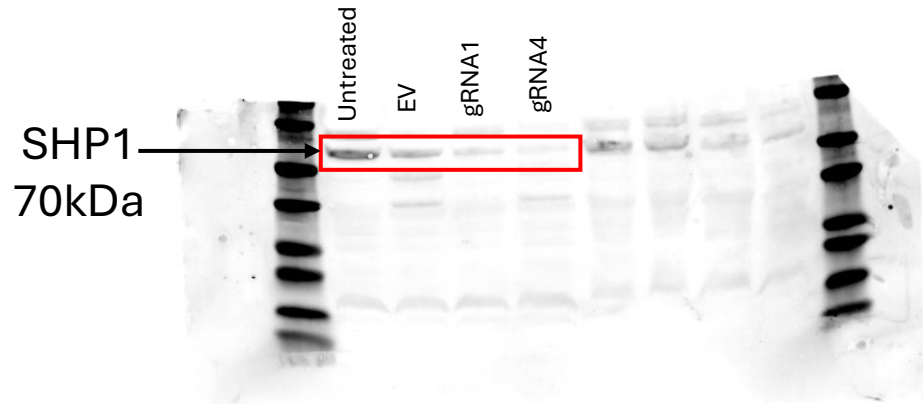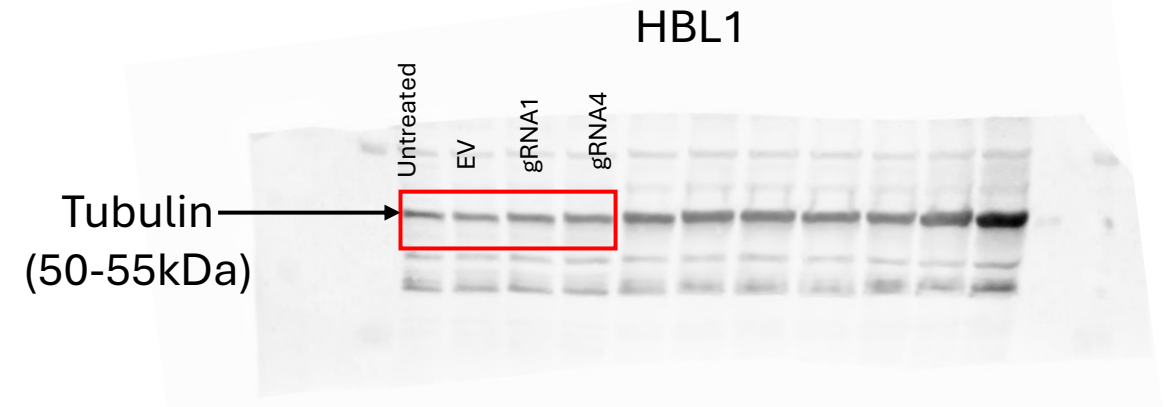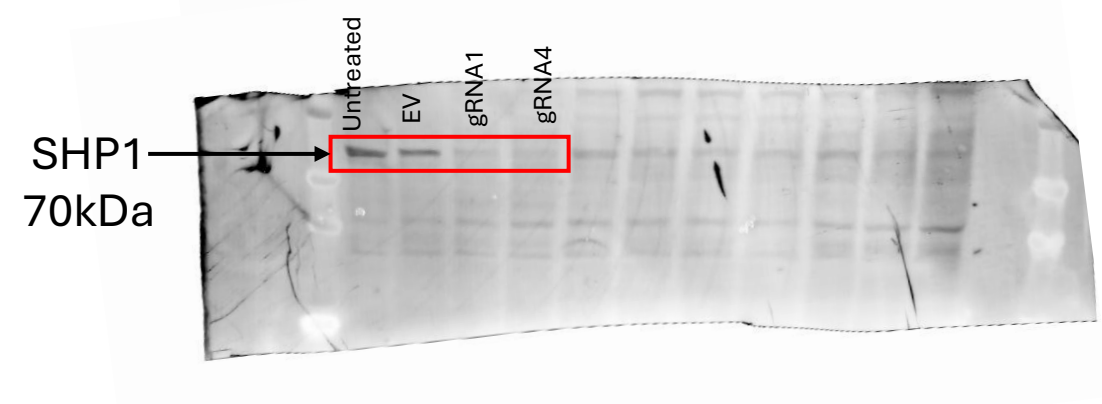

Supplement: Figure 5—figure supplement 1—source data 1. [file elife-106425-fig5-figsupp1-data1.zip › Figure 5 - figure supplement 1A - Source data 1/Figure 5 - figure supplement 1A.pdf]

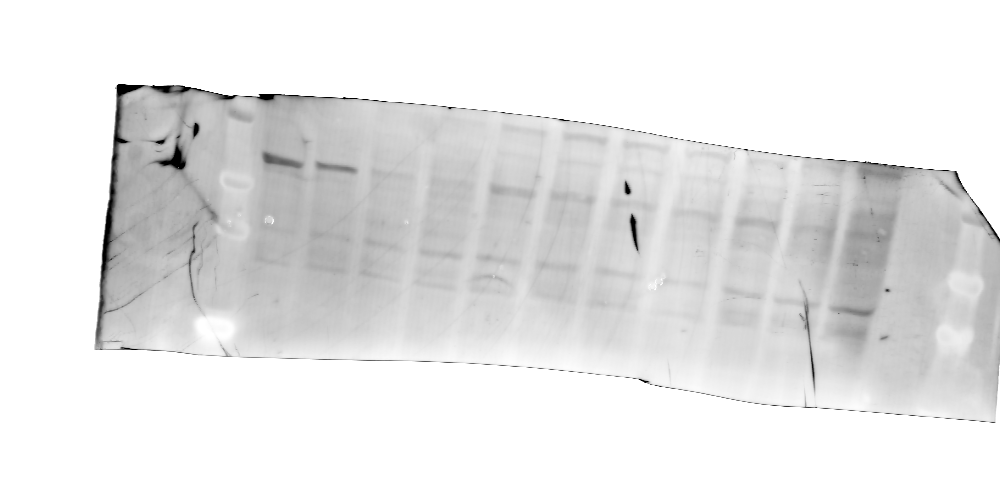

Supplement: Figure 5—figure supplement 1—source data 2. [file elife-106425-fig5-figsupp1-data2.zip › Figure 5 - figure supplement 1A-Source data 2/HBL1 SHP1[IRshort].tif]

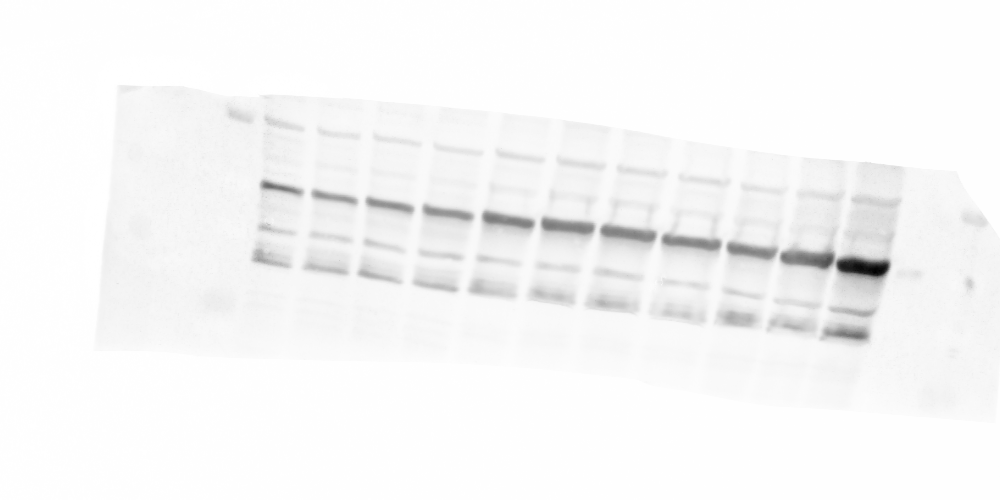

Supplement: Figure 5—figure supplement 1—source data 2. [file elife-106425-fig5-figsupp1-data2.zip › Figure 5 - figure supplement 1A-Source data 2/HBL1 Tubulin[IRlong].tif]

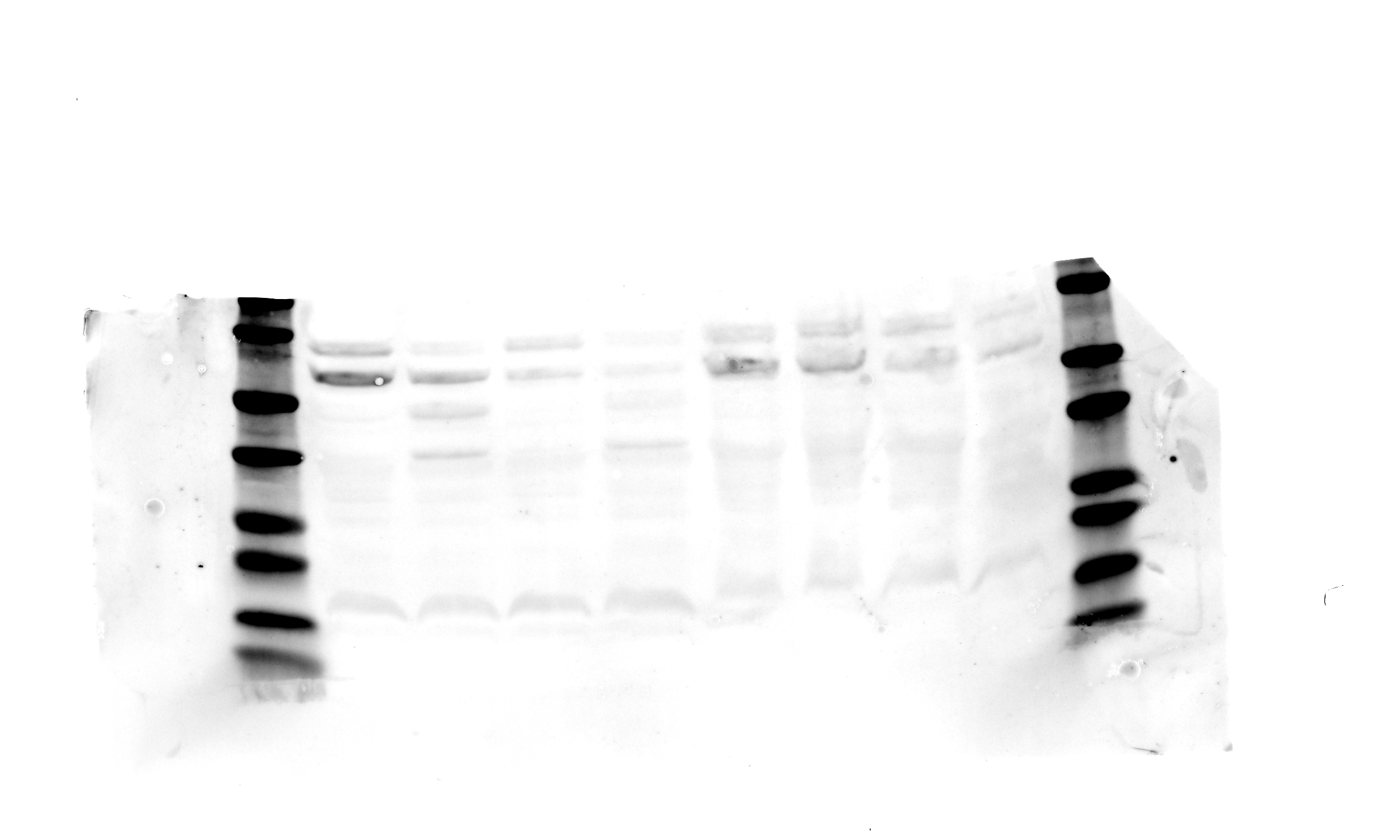

Supplement: Figure 5—figure supplement 1—source data 2. [file elife-106425-fig5-figsupp1-data2.zip › Figure 5 - figure supplement 1A-Source data 2/Oci-Ly7 SHP1[IRshort].tif]

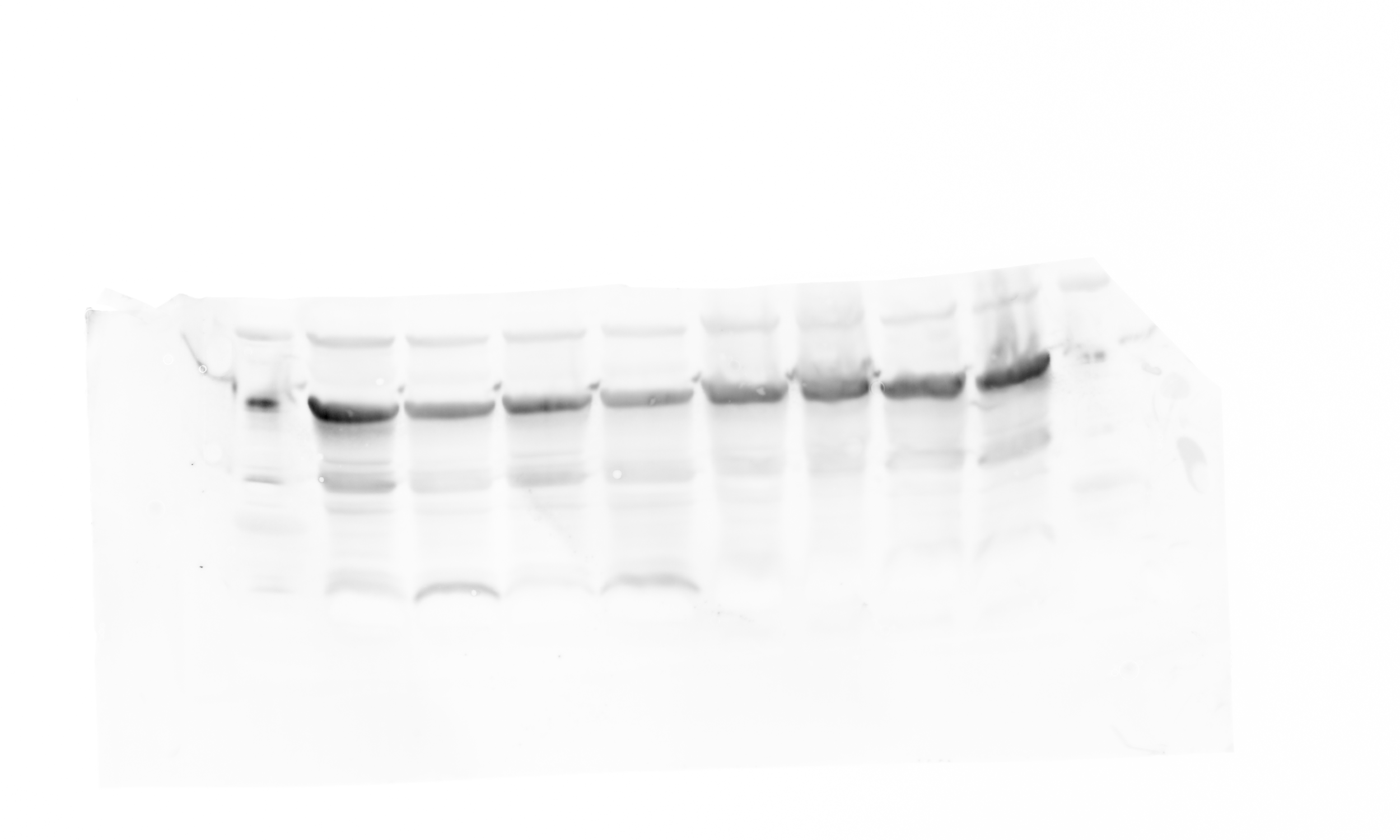

Supplement: Figure 5—figure supplement 1—source data 2. [file elife-106425-fig5-figsupp1-data2.zip › Figure 5 - figure supplement 1A-Source data 2/Oci-Ly7 Tubulin[IRlong].tif]
