# Supplementary material for: DuoHexaBody-CD37 induces direct cytotoxic signaling in diffuse large B-cell lymphoma
Source: eLife. 2026 Jul 20;14:RP106425. doi: 10.7554/eLife.106425 (PMC13384490; doi:10.7554/eLife.106425)
Supplement: Peer review history

# Editor report: eLife Assessment

**DOI:** 10.7554/eLife.106425.3.sa0

**Authors:** Piers Patten

**Affiliations:** King's College London United Kingdom

**Keywords:** Solid, Valuable

This study presents a **valuable** finding on the direct cytotoxic effects of DuoHexaBody-CD37 in diffuse large B-cell lymphoma through antibody clustering, independent of complement. The central findings are supported by **solid** evidence, although some mechanistic details, including the specific Fc receptor requirements for crosslinking-mediated cytotoxicity, remain unresolved. As the findings are based primarily on in vitro models, further validation would be required to support broader translational conclusions. The previous review comments were addressed by the authors and have improved the work.

# Referee report: Joint Public Review

**DOI:** 10.7554/eLife.106425.3.sa1

[Editor's Note: The previous reviewers comments were felt to be addressed by the reviewers and myself and have improved the work.]

In this study, the authors suggest that DuoHexaBody-CD37, a biparatopic CD37-targeting antibody, can induce direct cytotoxicity in diffuse large B-cell lymphoma (DLBCL) cells through antibody clustering and SHP-1 activation, independent of complement. They further propose that DuoHexaBody-CD37 inhibits cytokine-mediated pro-survival signalling, suggesting a broader role for CD37-directed therapy in disrupting tumour supportive signalling networks.

A strength of the study is the systematic in vitro characterisation of signalling responses to DuoHexaBody-CD37 across both malignant and normal B-cells. The inclusion of phosphoproteomic profiling and mutant constructs provides mechanistic detail, and the findings may be of interest to researchers working on antibody therapeutics in lymphoma.

However, the evidence supporting key mechanistic processes - particularly the specific subtype requirement for Fc receptor crosslinking - is incomplete and would benefit from further functional validation. While CD37 has been explored previously as a therapeutic target, this study does add mechanistic insight into direct cytotoxicity and cytokine modulation. Nevertheless, the exclusive reliance on in vitro systems makes the translational relevance unclear.

Overall, the study provides valuable insight into CD37-mediated signalling in lymphoma cells, but the evidence remains incomplete to support broader conclusions about therapeutic impact. The additional mechanistic data included during revision are informative, but the precise basis of the observed cytotoxic effects remains incompletely defined.

# Author comment: Author response

**DOI:** 10.7554/eLife.106425.3.sa2

**Authors:** Simar Pal Singh, Kumar Mangalam, Michelle D van den Beukel, Sjoerd van Deventer, Marije B Overdijk, Guy Roukens, Kim CM Santegoets, Esther CW Breij, Martin ter Beest, Willem PJ Cox, Annemiek B van Spriel

**Affiliations:** Radboud University Medical Center Nijmegen Netherlands; Radboud University Medical Center Nijmegen Netherlands; Radboud University Medical Center Nijmegen Netherlands; Radboud University Medical Center Nijmegen Netherlands; Genmab Utrecht Netherlands; Genmab Utrecht Netherlands; Genmab Utrecht Netherlands; Genmab Utrecht Netherlands; Radboud University Medical Center Nijmegen Netherlands; Radboud University Medical Center Nijmegen Netherlands; Radboud University Medical Center Nijmegen Netherlands

The following is the authors’ response to the original reviews.

**Public Reviews:**

**Joint Public Review:**

In this study, the authors suggest that DuoHexaBody-CD37, a biparatopic CD37-targeting antibody, can induce direct cytotoxicity in diffuse large B-cell lymphoma (DLBCL) cells through antibody clustering and SHP-1 activation, independent of complement. They further propose that DuoHexaBody-CD37 inhibits cytokinemediated pro-survival signalling, suggesting a broader role for CD37-directed therapy in disrupting tumour supportive signalling networks.

A strength of the study is the systematic in vitro characterisation of signalling responses to DuoHexaBodyCD37 across both malignant and normal B-cells. The inclusion of phosphoproteomic profiling and mutant constructs provides mechanistic detail, and the findings may be of interest to researchers working on antibody therapeutics in lymphoma.

However, the evidence supporting key mechanistic processes - particularly the role of SHP-1 in mediating cytotoxicity and the requirement for Fc receptor crosslinking - is incomplete and would benefit from further functional validation. While CD37 has been explored previously as a therapeutic target, this study does add mechanistic insight into direct cytotoxicity and cytokine modulation. Nevertheless, the exclusive reliance on in vitro systems makes the translational relevance unclear. Overall, the study provides valuable insight into CD37-mediated signalling in lymphoma cells, but the evidence remains incomplete to support broader conclusions about therapeutic impact.

**Recommendations for the authors:**

**Reviewer #1 (Recommendations for the authors):**

In the manuscript, Singh and colleagues reveal a new mechanism via which DuoHexaBody-CD37 induces DLBCL cytotoxicity, which is independent of external factors, such as the effector cells and the complement system. As cited by the authors, the induction of B cell death has previously been demonstrated for antibodies directed against B cells, including anti-CD37 (otlertuzumab). Furthermore, the majority of these observations are made using in vitro systems, and it is not clear if this phenomenon happens in vivo or not?

Thank you for pointing this out. We would like to refer to previous report that have demonstrated potent anti-tumor activity of DuoHexaBody-CD37 in vivo in cell line- and patient-derived xenograft models from different B-cell malignancy subtypes [PMID: 32341336]. Moreover, DuoHexaBody-CD37 ex vivo activity has been shown in primary tumor cell samples from a large cohort of newly diagnosed (ND) and relapsed/refractory (RR) patients with a broad range of B-cell malignancies, including chronic lymphocytic leukemia (CLL) and B-cell non-Hodgkin lymphoma, including diffuse large B-cell lymphoma (DLBCL) [PMID: 33324950]. We refer to these data in the introduction.

The presented data suggest that DuoHexaBody-CD37 relies on Fc crosslinking for its optimal cytotoxic activity. Investigating which FcγR is needed for this purpose would have been useful, as FcγRIIb, for instance, has been shown to be important in supporting the therapeutic function of mAbs like anti-CD40.

We thank you for this suggestion. To further investigate the role of specific FcγRs in effector cell-mediated Fc cross-linking, PBMC-mediated direct cytotoxicity was compared across various immune cell subsets: B cells (FcγRIIb), NK cells (FcγRIIIa, IIc), monocytes (FcγRI, IIa/b, IIIb), and T cells (no confirmed FcγR expression). Notably, all immune cells subsets expressing FcγRs exhibited similar or enhanced cytotoxicity against DLBCL cells compared to the total PBMC pool. These results indicate that DuoHexaBody-CD37 induced killing is independent of specific FcγR subtypes. We have added these new data to new Figure 1C.

Specific comments:

(1) Line 92:93: The authors should also cite the following reference for rituximab: https://pubmed.ncbi.nlm.nih.gov/19620786/ .

We have added this reference to the revised paper (ref. 31).

(2) Figure 1 and 2: Since cell death was only observed in the presence of crosslinking in Figure 1, Figure 2 should also investigate the clustering and internalization of CD37 in the presence of the same secondary antibody. It is likely that DuoHexaBody-CD37 will induce receptor internalization upon crosslinking.

To further investigate internalization, we compared the surface availability of CD37 with and without Fc-mediated crosslinking of DuoHexaBody-CD37 across cell lines. Little to no decrease in the surface availability of CD37 upon Fc-mediated crosslinking (new Supplementary Figure 2) was observed.

In addition, we performed cluster analysis studies in lymphoma cells treated with DuoHexabody-CD37 in the absence and presence of Fc-crosslinking (and respective isotype controls). We observed that DuoHexabodyCD37 by itself was already sufficient to induce CD37 clustering, which was further enhanced by Fc-crosslinking (new Figure 2A, B).

(3) Figure 3A: the Y-axes should be clearly labelled.

Done.

(4) Figure 6: What is the reason for the selective use of different cell lines in Figure 6? Additionally, only 1 donor has been used for the IL-6 analysis.

The reviewer is indeed correct in noticing that only one cell line has been used for the IL-6 analysis. We observed that HBL-1 cells were the only cell line that were sensitive to IL-6 treatment, in contrast to IL-4 and IL-21. We have added this sentence to the discussion to explain this better: “p-STAT3 downregulation upon DuoHexaBody-CD37 treatment in presence of IL-6 requires further investigation in additional IL-6-responsive cell lines, as HBL1 was the only IL-6-responsive lymphoma cell line tested in this study.”

The data shown in Figure 6 are results from at least three independent experiments (each dot is an independent experiment, not a donor).

**Reviewer #2 (Recommendations for the authors):**

Singh et al uncover a novel mechanism of action for the DuoHexaBody-CD37 against DLBCL, whereby it is shown to induce direct cytotoxicity independent of complement and to activate the phosphatase SHP-1. DuoHexaBody-CD37 is also shown to reduce cytokine induced JAK/STAT signalling in DLBCL cells.

Strengths:

The authors provide novel insight into CD37 targeting across normal B cells, DLBCL and Burkitt lymphoma cells, which have the potential to inform clinical translation.

Weaknesses:

The mechanisms behind differences in signalling and apoptosis between normal B cells, Burkitt lymphoma, and DLBCL cells with CD37 targeting require further clarification. In particular, the contribution of SHP-1 to this effect is not clear and indeed is increased in both normal b cells and DLBCL cells.

Key points that require addressing are below:

(1) Viability of Burkitt lines was less affected than DLBCL in Figure 1- this should be compared with surface CD37 expression in these same lines to determine whether this accounts for the effect. This difference is a key finding for clinical translation.

We thank the reviewer for this suggestion and we have now performed flow cytometry analysis across DLBCL and Burkitt cell lines upon staining with two different anti-CD37 antibodies (WR17, M-B371) to quantify membrane CD37 expression (new Supplementary Figure 1B). These data show that CD37 expression levels are not directly related to DuoHexaBody-CD37 mediated cytotoxicity in the studied B cell lines.

(2) pSHP1 is increased in both normal B cells (lines 169-171, Figure 3C) and DLBCL and yet the authors state specific upregulation of pSHP1 in DLBCL as a reason for induced cytotoxicity in DLBCL (lines 183-185). This requires clarification and experimental confirmation. The authors should investigate normal B cells in the cytotoxicity assays as in Figure 1 for comparison. The authors should also confirm the importance of SHP-1 in this apoptosis process using specific SHP pharmacological agents, which are commercially available.

To analyze the role of SHP1 mediated signaling in induced cytotoxicity of DLBCL, SHP1 knock outs (KO) were generated in HBL1 and OciLy7 cell lines using CRISPR Cas9 technology (new Supplementary figure 5A). The wild type and SHP-1 KO cell lines were then compared for differences in cytotoxicity after treatment with DuoHexaBody-CD37 with and without Fc-crosslinker. No differences in cytotoxicity were observed between the wild type and knock out cell lines (new Supplementary figure 5B), indicating that DuoHexaBody-CD37induced SHP1 signaling does not play a direct role in the increased cytotoxicity. We have added these new data to the results and rephrased the role of SHP-1 in the revised manuscript.

(3) It would be informative to assess caspase activation and PARP cleavage across normal B cells, DLBCL and Burkitt under these conditions for clarity on apoptosis induction.

We thank the reviewer and we agree it would be informative to confirm apoptosis induction in the cell lines upon DuoHexaBody-CD37 treatment. We addressed this question by flow cytometric analysis of different lymphoma cell lines stained with/without Annexin V (apoptosis marker) and 7AAD (late apoptotic/necrotic marker) in presence or absence of DuoHexaBody-CD37, with and without Fc-crosslinking. These experiments demonstrate that Fc-crosslinking DuoHexaBody-CD37 leads to the induction of apoptosis across DLBCL cell lines (new Supplementary Figure 1A).

(4) The regulation of JAK/STAT signalling by SHP-1 should be mentioned in the introduction and discussion as this is a key finding of the manuscript.

Based on the new data on the role of SHP-1 (Suppl. Fig. 5), we have rephrased the text on the SHP1 in the discussion of the revised paper: “DuoHexaBody-CD37 treatment also led to an increase in SHP1 mediated signaling, however we could not confirm a direct role of SHP1 signaling in DuoHexaBody-CD37-mediated cytotoxicity. DLBCL cells may undergo signal rewiring upon SHP1 knockdown by altered levels of p‑AKT, p‑STAT3, and p‑STAT6, or SHP2 may compensate for the loss of SHP1. It is currently unclear what the biological implications are of the increased SHP1 signaling observed upon treatment with DuoHexaBody-CD37 in DLBCL cells.”

(5) The authors state that DuoHexabody-37 is particularly effective at downregulating STAT signalling in the presence of IL-6 (lines 302-303) however, this is not statistically significant in the results section. There is a trend for a reduction, however, further experimental repeats would clarify this.

We agree with the reviewer, and rewrote the text on IL-6 in the discussion: “p-STAT3 downregulation upon DuoHexaBody-CD37 treatment in presence of IL-6 requires further investigation in additional IL-6-responsive cell lines, as HBL1 was the only IL-6-responsive lymphoma cell line tested in this study.”
